# A tet-on system for Drd1a-expressing cells

**DOI:** 10.1186/1750-1326-7-S1-O13

**Published:** 2012-02-07

**Authors:** Fayi Yao, Robert G MacKenzie

**Affiliations:** 1 Dept Psychiatry and Behavioral Neurosciences, Wayne State University School of Medicine, Detroit, Michigan 48201, USA

## Background

Neurons of the striatum receive important glutaminergic input from the cortex and thalamus and massive dopaminergic innervation from midbrain dopamine neurons. The majority (95%) of striatal neurons are medium spiny projection neurons and 5% are aspiny interneurons. The projection neurons are GABAergic and form the direct and indirect striatal output pathways that have been shown to be crucial for normal motor function and appetitively motivated learning. The importance of the medium spiny projection neurons is highlighted by the severe debilitation associated with the loss of their dopaminergic input in Parkinson’s disease or degeneration of these cells in Huntington’s disease (Kreitzer, Ann Rev Neurosci, 32, 127-147, 2009). Approximately half of all medium spiny striatal neurons express the dopamine D1A receptor (Drd1a) and these neurons comprise the direct output pathway to the medial globus pallidus and substantia nigra pars reticulata (Valjent et al., TINs, 32, 538-547, 2009). Early deletion of the Drd1-expressing striatal neurons in mice results in severe motor impairment, eating deficits and reduced striatal volume (Drago, et al., J Neurosci, 18, 9845-9857, 1998). In order to separate potential developmental effects from the normal role of the Drd1a-expressing striatal cells in adult mice, we have made a mouse model that allows for the inducible deletion or expression of targeted genes specifically within the Drd1a-expressing striatal neurons using BAC transgenesis.

## Methods

A bacterial artificial chromosome (BAC) designated RP23-47M2 was obtained from BACPAC resources of the Children’s Hospital Oakland Research Institute (CHORI). This BAC contains the Drd1a locus. Using the techniques of bacterial homologous recombination (Liu, et al., Genome Res, 13, 476-484, 2003), a cassette containing the reverse tetracycline-controlled transactivator (rtTA) fused to the herpes simplex virus VP16 transactivator was inserted at the ATG start site of the Drd1a locus. Transgenic mice with the modified BAC randomly integrated into their genomes were produced by microinjection of the modified BAC into mouse zygotes at the University of Michigan Transgenic Core. Transgenic mouse lines carrying the rtTA allele were crossed with reporter transgenic mice carrying the allele for tetO-lacZ. In bi-transgenic mice this approach (TET-ON) allows for the activation of the tet operator by rtTA in the presence of doxycycline (DOX). Therefore, during DOX administration, the lacZ gene produces β-galactosidase which can be detected through X-gal staining.

## Results

Drd1a-rtTA/tetO-lacZ lines were screened and at least one line to date has shown strong expression specifically within the mouse striatum. X-gal staining is present throughout the striatum, with somewhat higher levels in the medial aspects of the structure. We are continuing to screen additional lines and are determining the minimal parameters of DOX administration (dose and duration) necessary for the inducible expression of rtTA/VP16.

## Conclusion

We have made the first inducible Drd1a TET-ON mouse. This mouse, in conjunction with other transgenic models, will allow for the inducible expression or deletion of target genes specifically within Drd1a-expressing cells and will be highly useful in the study of Parkinson’s and Huntington’s diseases, addiction and the normal role of Drd1a-expressing cells.

